# Loss of OxyR reduces efficacy of oxygen respiration in *Shewanella oneidensis*

**DOI:** 10.1038/srep42609

**Published:** 2017-02-14

**Authors:** Fen Wan, Miaomiao Shi, Haichun Gao

**Affiliations:** 1 Institute of Microbiology and College of Life Sciences, Zhejiang University, Hangzhou, Zhejiang, 310058, China

## Abstract

In many bacteria, OxyR is the major regulator controlling cellular response to H_2_O_2_. A common phenotype resulting from OxyR loss is reduced growth rate, but the underlying mechanism is unknown. We demonstrated in *Shewanella oneidensis*, an important research model for applied and environmental microbes, that the defect is primarily due to an electron shortage to major terminal oxidase cytochrome *cbb*_3_. The loss of OxyR leads to enhanced production of electron carriers that compete for electrons against cytochrome *cbb*_3_, cytochrome *bd* in particular. We further showed that the *oxyR* mutation also results in increased production of menaquinone, an additional means to lessen electrons to cytochrome *cbb*_3_. Although regulation of OxyR on these biological processes appears to be indirect, these data indicate that the regulator plays a previously underappreciated role in mediating respiration.

Oxidative stress, caused by reactive oxygen species (ROS), including superoxide (O_2_^−^), hydrogen peroxide (H_2_O_2_), and hydroxyl radical (OH), is one of the unavoidable crises for the vast majority of aerobic organisms by damaging biomolecules such as lipids, proteins and DNA^1^. ROS (H_2_O_2_ in particular) are ubiquitous because they are metabolic by-products of cellular oxygen respiration, mostly through the accidental auto-oxidation of non-respiratory flavoproteins and the turnover of committed oxidases, in addition to those coming from environments^2^,^3^,^4^. To cope with oxidative stress, bacteria have evolved multiple protection systems, such as ROS detoxification enzymes (alkylhydroperoxide reductase (Ahp), catalases, and various peroxidases), iron-sequestering proteins (Dps in particular), and oxidative damage repairing macromolecules^5^. When ROS levels exceed safe limits, in response these systems are induced coordinately by regulators to ensure that the ROS concentrations are restrained at an acceptable level and damages are promptly repaired. In *Escherichia coli*, the model organism in which the oxidative stress response is best understood, and many other bacteria, SoxRS and OxyR, are the predominant regulatory systems mediating cellular response to O_2_^−^ (redox-cycling compounds) and H_2_O_2_, respectively^1^. OxyR, a LysR family transcriptional regulator, directly senses H_2_O_2_ via its two conserved cysteine residues that react rapidly with H_2_O_2_. In the presence of H_2_O_2_ that is sufficient to mount the response, these residues are oxidized and an intramolecular disulfide bond is formed, leading to OxyR activation, which subsequently turns on expression of the defending systems. However, exceptions to the *E. coli* OxyR model are found. In *Neisseria*, OxyR acts as both a repressor of catalase expression and an activator for other members of its regulon^6^.

*Shewanella*, a group of Gram-negative facultative γ-proteobacteria, inhabit redox-stratified environments and are renowned for their versatile respiratory abilities, which underlies great potential for bioremediation and microbial fuel cells^7^. These bacteria are substantially more sensitive to H_2_O_2_ than *E. coli*, in concert with the high susceptibility to UV and ionizing radiation, understandings primarily derived from studies on the model species *S. oneidensis*^8^,^9^,^10^,^11^. *S. oneidensis* OxyR appears to be the only possible regulator mediating the cellular response to both O_2_^−^ and H_2_O_2_ because it lacks a homologue of *E. coli* SoxRS^12^,^13^. *S. oneidensis* OxyR acts as both an activator and a repressor as *Neisseria* OxyR proteins^6^; upon OxyR activation, *S. oneidensis* mainly relies on derepression of *katB* (encoding the major catalase) and *dps* genes to alleviate oxidative damage by degrading H_2_O_2_ and by sequestering the intracellular free iron, respectively^12^. As a result, an *S. oneidensis oxyR* mutant degrades H_2_O_2_ more rapidly than the wild-type. Despite this, the *oxyR* mutant bears defects in both viability (plating defect) and growth, which are commonly observed in bacteria whose OxyR proteins act as a positive regulator for catalase genes, such as *E. coli*^12^,^13^,^14^,^15^.

Oxygen respiration is carried out by respiratory oxygen reductases (traditionally dubbed as terminal oxidases)^16^. Among them, heme-copper oxidases (HCOs) and cytochrome *bd* are found in a wide variety of prokaryotes. While HCOs generate a proton motive force (PMF) via a highly efficient “proton pumping” mechanism, cytochrome *bd* does not and therefore is characterized by a lower energetic efficiency^17^. The *S. oneidensis* genome encodes three terminal oxidases: a *bd*-type quinol oxidase (*cydABX*) and two heme-copper oxidases (HCOs), a *caa*_3_-type (*SO4606-9*) and a *cbb*_3_-type (*ccoNOQP*)^18^,^19^,^20^. Cytochrome *cbb*_3_ is abundant in membranes under both aerobic and microaerobic conditions whereas cytochrome *caa*_3_ is not present; thus cytochrome *cbb*_3_ is the predominant force for oxygen respiration^18^,^21^. Cytochrome *bd*, like its *E. coli* counterpart, plays a minor role in oxygen respiration, and more importantly, confers *S. oneidensis* tolerance to various stresses, especially nitrite^22^,^23^,^24^. Consequently, the loss of cytochrome *cbb*_3_ but not cytochrome *bd* leads to a significant defect in growth under aerobic conditions^18^. All terminal oxidases, either directly or via soluble cytochrome *c*, accept electrons from quinones (UQs), which are the most important compounds in the membrane of most prokaryotes, working as lipophilic electron and proton shuttles^16^,^25^. In *S. oneidensis*, quinones include ubiquinones (UQs) and menaquinones (MKs); MKs, such as MK-7 (−80 mV), have low midpoint redox potentials compared with those of UQs, such as UQ-8 (+110 mV)^26^,^27^,^28^. While cytochrome *cbb*_3_ (via cytochrome *bc*_1_) and cytochrome *bd* predominantly use UQs as the electron donor MKs supply electrons to terminal reductases for many non-oxygen EAs with CymA working as quinone oxidase^29^,^30^,^31^. Interestingly, in batch cultures under aerobic conditions, MKs are still substantial in abundance, accounting for slightly less 50% of the quinone composition, one of features underlying its respiratory diversity^27^,^29^,^32^.

Although most, if not all, of *oxyR* mutants studied to date carry growing defect, the underlying mechanism remains elusive. Previously we have shown that the plating defect of the *S. oneidensis oxyR* mutant is primarily due to H_2_O_2_ that is generated abiotically, providing intriguing insights into current understandings of OxyR physiology^10^,^13^. The goal of this study was to address biological factors that are responsible for the growing defect of bacteria, with *S. oneidensis* as research model. We demonstrated that this defect is independent of the plating defect. By using a random mutational analysis, we identified cytochrome *bd* to be associated with the phenotype. We then showed evidence to suggest that cytochrome *bd*, as well as CymA, hampers growth by diminishing the quantity of electrons to cytochrome *cbb*_3_. Furthermore, we found that the *oxyR* mutation also results in increased production of menaquinone-7, which acts as additional means to lessen electrons to cytochrome *cbb*_3_.

## Methods

### Bacterial strains, plasmids and culture conditions

All bacterial strains and plasmids used in this study can be found in Table 1. Information about all of the primers used in this study was available upon request. All chemicals were obtained from Sigma-Aldrich unless otherwise noted. *E. coli* and *S. oneidensis* were grown in Lysogeny broth (LB, Difco, Detroit, MI) under aerobic conditions at 37 and 30 °C for genetic manipulation. When necessary, the growth medium was supplemented with chemicals at the following concentrations: 2,6-diaminopimelic acid (DAP), 0.3 mM; ampicillin, 50 μg/ml; kanamycin, 50 μg/ml; and gentamycin, 15 μg/ml; streptomycin, 100 μg/ml; and catalase on plates, 2000 U/ml.

Growth in liquid media was monitored by recording values of optical density at 600 nM (OD_600_) as all strains used in this study were morphologically similar. Both LB and defined medium MS^13^ were used for phenotypic assays in this study and comparable results with respect to growth were obtained.

### In-frame mutant construction and complementation

In-frame deletion strains for *S. oneidensis* were constructed using the *att*-based Fusion PCR method as described previously^33^. In brief, two fragments flanking gene of interest were amplified by PCR with primers containing *attB* and the gene specific sequence, which were linked by a second round of PCR. The fusion fragments were introduced into plasmid pHGM01 by using Gateway BP clonase II enzyme mix (Invitrogen) according to the manufacturer’s instruction. The resultant plasmids were transformed by electroporation into and maintained in *E. coli* WM3064, and transferred to *S. oneidensis* by conjugation with the ratio of the donor to the recipient being 2:1. Integration of the mutagenesis constructs into the chromosome was selected by resistance to gentamycin and confirmed by PCR. Verified transconjugants were grown in LB broth in the absence of NaCl and plated on LB supplemented with 10% sucrose. Gentamycin-sensitive and sucrose-resistant colonies were screened by PCR for deletion of the target gene. Mutants were verified by sequencing the mutated regions.

Promoterless plasmid pHG101 was used in genetic complementation of mutants as described before^34^. For inducible gene expression, the gene of interest was generated by PCR and introduced into the inducible plasmid pHGE-Ptac under the control of promoter P*tac*^35^. After sequencing verification, the resulting vectors were transferred into the relevant strains via *E. coli* WM3064-mediated conjugation.

### Transposon mutagenesis

Random mutagenesis vector pHGT01, which carries a robust promoter in the transposable region, was used to construct a library in this study^20^. Briefly, the vector from *E. coli* WM3064 was transferred into *S. oneidensis* Δ*oxyR* via conjugation and growth suppressor strains were screened on plates containing gentamycin and catalase. Plates having 200–300 colonies were analyzed by a specific program to calculate the average size and size ratios of each colony to the average. The colonies, whose size was similar to that of the wild-type, were saved and subsequently subjected to the determination of the transposon insertion sites using the arbitrary PCR^36^.

### Expression analysis

Expression of genes of interest was determined by β-Galactosidase activity assay using integrative *lacZ* reporter pHGEI01^32^. In brief, the sequence in sufficient length (~400 bp) upstream of gene of interest was amplified and placed in front of the full-length *E. coli lacZ* gene. The resulting vector was transformed into and maintained in *E. coli* WM3064, and transferred to *S. oneidensis* via conjugation after verification by sequencing. Cells at the mid-log phase (~0.3 of optical density at 600 nM (OD_600_)) were harvested by centrifugation, washed with phosphate-buffered saline (PBS, pH 7.0) and lyzed with the lysis buffer (0.25 M Tris/HCl, 0.5% Triton X-100, pH 7.5). Soluble proteins were collected after centrifugation and applied to the o-nitrophenyl-β-D-galactopyranoside (ONPG)-based assay as described previously^37^. The protein concentration of the cell lysates was determined using a Bradford assay with BSA as a standard (Bio-Rad). β-galactosidase activity were determined by monitoring color development at 420 nm using a Synergy 2 Pro200 Multi-Detection Microplate Reader (Tecan), presented as Miller units.

### Droplet assays

Droplet assays were employed to evaluate viability and growth inhibition on plates essentially the same as previously described^12^,^22^. In brief, cells at the mid-log phase were collected by centrifugation and adjusted to 10^9^ cell/ml, which was set as the undiluted (dilution factor 0). 10-fold serial dilutions were prepared with fresh LB medium. Five micro liters of each dilution was dropped onto LB plates, on which designated agents (catalase and nitrite) may be added. The plates were incubated for 24 h or longer in dark before being read. All experiments were repeated at least three times.

### Cytochrome oxidase activity assay

The Nadi test was used for visual analysis of cytochrome *cbb3* activity^38^. Five micro liters of each culture at the mid-log phase under test was dropped onto LB plates w/o catalase, and the plates were incubated for 24 h. A solution of 0.5% *α*-naphthol in 95% enthanol and 0.5% N,N-dimethyl-*ρ*-phenyleneidiamine monohydrochloride (DMPD) was applied to cover the droplets developed. Formation of indophenols blue was timed as an indicator of cytochrome *cbb3* activity.

Solubilized membranes were prepared for quantitative analysis of the cytochrome oxidase activity as described previously^20^. In brief, cell pellets were resuspended in 20 mM Tris-HCl (pH 7.6) supplemented with DNase I and protease inhibitors and disrupted by French pressure. After debris and unbroken cells removing, the membranes were pelleted by ultracentrifugation for 1 h at 230,000 × g at 4 °C and subsequently resuspended in 20 mM Tris-HCl pH 7.6 with 5% glycerol to a protein concentration of 10 mg/ml. Solubilization was performed with *n*-dodecyl β-D-maltoside (DDM) to a final concentration of 1% (w/v) on a rotary tube mixer for 2 h at 4 °C. The DDM-solubilized membranes were obtained by collecting the supernatant after ultracentrifuging for 1 h at 230,000 × g at 4 °C. The cytochrome oxidase activity was assayed as a measure of oxygen consumption rates using an OxyGraph oxygen electrode (Hansatech) using either ubiquinol-1 or *N,N,N*′,*N*′-tetramethyl-*p*-phenylenediamine (TMPD) as electron donor according to the methods described previously^20^.

#### Determination of oxygen consumption and NAD^+^/NADH ratio

For determination of oxygen consumption, a 2-mm O_2_ sensor and a TBR4100 free radical analyzer (World Precision Instruments) were used. Cells were washed three times in 33 mM potassium phosphate buffer (pH 7.0) and adjusted to an OD_600_ of 1. Oxygen consumption was induced by addition of either 100 mM disodium succinate as the electron donor. The basal oxygen consumption in the absence of the electron donor was subtracted; oxygen consumption is indicated as nmol oxygen ml^−1^ min^−1^.

The ratio of NAD^+^ to NADH was determined with a NAD/NADH quantitation kit (Sigma-Aldrich). *S. oneidensis* cultures of the mid-log phase were collected by centrifugation, washed with cold PBS, resuspended in the extraction buffer, and disrupted by sonication (three cycles of 2 min, on/off at 1-s intervals, at 50 W) on ice. After centrifugation at 20,000 × *g* for 5 min at 4 °C, the supernatants were deproteinated using 3,000-molecular-weight-cutoff (MWCO) spin filters (Millipore Corporation, Bedford, MA), and then subjected to the NAD^+^/NADH ratio determination according to the manufacturer’s instructions.

### Quinone extraction and quantification

Quinone extraction was performed on cultures at the mid-log phase with the methanol and petroleum ether method^32^,^39^. The extracted quinone-quinol mixture was resuspended with a glass rod in 80 μl ethanol and fractionated by HPLC (Thermo Accela UHPLC) with a Phenomenex reverse phase C18 (5 μm) column (dimensions 150 × 4.6 mm) with ethanol/methanol (50/50 v/v) as the mobile phase at 0.3 or 0.5 ml min^−1^. Detection of the quinones was performed at 290 nm for UQs and at 248 nm for MKs. The amount of each quinone species was calculated from the related peak area, using ubiquinone-10 (UQ_10_) amd menaquinone-4 (MK_4_) as standards. Peaks were identified by mass spectral analysis as described previously^40^. Fractions collected from the HPLC were evaporated under nitrogen and redissolved in 90% (wt/vol) acetonitrile, 1% (vol/vol) formic acid, analyzed by using a high solution, high-accuracy mass spectrometer (MS) (Exactive, ThermoFisher).

### Site-directed mutagenesis

Plasmid pTRG-OxyR was used as the template for site-directed mutagenesis with a QuikChange II XL site-directed mutagenesis kit (Stratagene) as described previously^40^. The resulting pTRG-OxyR^L197P^ was verified by sequencing.

### Bacterial one-hybrid (B1H) assay

B1H system (Stratagene) was used to investigate DNA-protein interaction *in vivo* in *E. coli* cells^12^,^41^. Briefly, plasmid constructs were created by cloning the bait DNA (promoter sequence of interest) and target DNA (the *oxyR* gene) in the pBXcmT and pTRG vectors and verified by sequencing. The resultant plasmids were used to co-transform BacterioMatch II Validation Reporter Competent Cells on M9 salt agar plates containing 25 mg/ml chloramphenicol and 12.5 mg/ml tetracycline with or without 3-amino-1,2,4-triazole (3-AT). Pairs of pBXcmT-P_*katB*_/pTRG-OxyR and pBXcmT-P_*acpC*_/pTRG-OxyR^L197P^ were used as positive control and a ~300 bp DNA fragment of the 16 s rRNA gene promoter (pBXcmT-P_*16s-rRNA*_) was used as negative control. The plates were incubated for 24 h and then moved to room temperature for an additional 16 h (the colonies indicating positive interaction usually appeared between 18 and 24 h). The positive interactions were confirmed by streaking colonies on plates containing both 3-AT and streptomycin (12.5 mg/ml).

### Statistical analyses

Values are presented as means ± SD (standard deviation). In some cases, error bars, which represent SD and less than 10% of the average of experimental values, were omitted for clarity. Student’s *t*-test was performed with statistical significance set at the 0.05 confidence level.

## Results

### Defects of Δ*oxyR* in growth and viability are independent of each other

**Figure 1:**
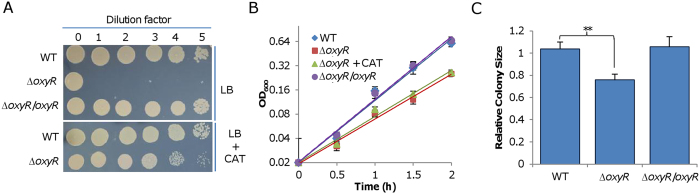
The growth defect of the *oxyR* mutant is independent of the plating defect. (**A**) Plating phenotype of indicated strains. Cultures at the mid-log phase were collected and adjusted to 10^9^ cell/ml (dilution factor 0), which were then serially diluted and 5 μl of each dilution was dropped onto indicated agar plates. Results shown were after growth of the wild-type in the lowest cell density (10^4^ cell/ml, dilution factor 5) became evident. (**B**) Growth of indicated strains in LB without or with catalase (CAT), including the wild-type (WT), ∆*oxyR*, genetically complemented ∆*oxyR* (∆*oxyR*/*oxyR*). OD_600_ values of the five time points during the exponential phase were plotted, with exponential trendlines (representing growth rate) being displayed (the same thereafter). (**C**) Growth of indicated strains on LB plates containing CAT. Cultures at the mid-log phase were serially diluted and spread onto LB plates containing CAT. After 24 h, plates having ~100 colonies were photographed and the averaged size of 21 colonies on (7 per plate) three plates for each strain was calculated out. Presented was the ratio of the averaged sizes of mutants to that of the wild-type. **p < 0.01. Experiments were performed at least three times, with representative results and the average ± error bars representing standard deviation being presented.

The plating defect of the *S. oneidensis oxyR* mutant is due to H_2_O_2_ generated abiotically on LB plates^12^,^13^. On LB agar plates, only undiluted Δ*oxyR* culture can grow (Fig. 1A, dilution factor 0, ~10^9^ cell/ml). In LB broth, while the viability defect of Δ*oxyR* is not evident because of catalase KatB released from lyzed cells^10^, significant difference in growth rates between the wild-type and Δ*oxyR* strains was observed (Fig. 1B). The growth defect in LB broth can be fully corrected by a copy of the *oxyR* gene expressed *in trans*, but was barely affected by catalase added to levels (2000 U/ml) that were sufficiently high to rescue the plating defect. We then examined effect of catalase on growth of the Δ*oxyR* strain on plates. Consistently, colony sizes of the wild-type and the Δ*oxyR* strains differed significantly in the presence of catalase (Fig. 1C). After 24 h, colonies of the Δ*oxyR* strain were approximately 24% smaller than those of the wild-type, ruling out the possibility that H_2_O_2_ generated abiotically is also the cause for the growing defect of the Δ*oxyR* strain. We then performed the same experiment with superoxide dismutase (SOD) to examine whether superoxide has a role in these two phenotypes. However, the addition of SOD had no effect on alleviating either the plating or growing defect of the Δ*oxyR* strain (data not shown). These data, all together, suggest that mechanisms for the plating and growing defects of the Δ*oxyR* strain are distinct.

### Loss of cytochrome *bd* suppresses the growth defect of the *oxyR* mutant

**Figure 2:**
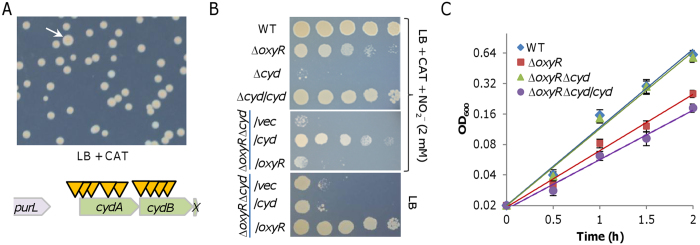
Cytochrome *bd* is likely associated with the growth defect of the *oxyR* mutant. (**A**) Suppressors for growth defect of ∆*oxyR*. Shown were representatives of stable suppressor strains derived from ∆*oxyR* on CAT-containing LB plates. Schematics indicating the approximate locations of the transposon insertions were shown below. Arrows represent transposon insertion points. X represents *cydX*. (**B**) Nitrite sensitivity assay. Mid-log phase cultures were subject to 10-fold serial dilution, and 5 μl of each dilution was spotted onto plates containing 2 mM nitrite and CAT. Double mutant ∆*oxyR*∆*cyd* was complemented by either of the missing genes (∆*oxyR*∆*cyd*/*oxyR* and ∆*oxyR*∆*cyd*/*cyd*) on indicated plates to confirm effects of the additional loss of Cyd. (**C**) Growth of indicated strains in LB without or with catalase (CAT), including the wild-type (WT), ∆*oxyR*, ∆*oxyR*∆*cyd*, and genetically complemented ∆*oxyR*∆*cyd* (∆*oxyR*∆*cyd*/*cyd*). Experiments were performed at least three times and either representative results or error bars representing standard deviation.

To search for genes whose products are related to the growing defect of the Δ*oxyR* strain, we employed transposon-based mutational screening. As the Δ*oxyR* strain grows significantly slower than the wild-type, we reasoned that we may be able to obtain suppressor strains with elevated growth rate. Vector pHGT01, which can be used for construction of transposon insertion libraries as well as for identification of cryptic operons because of an embedded promoter in the transposable sequence, was used in this study^20^. The resulting library was diluted properly (100–300 colonies per plate) and spread out on LB plates containing catalase and required antibiotics. A total of ~15,000 colonies on plates were screened and 323 mutants grew to significantly increased size (at least 15% larger than the average size of the colonies on plates) (Fig. 2A). Most of these (276 of 323), however, were not stable, growing into colonies varying in size during confirmation (by re-plated on the same plate), and were thus discarded. Among the remaining, 18 displaying largest colony size (indistinguishable from the wild-type) were subject to transposon insertion mapping. In 16 out of them, the transposon was successfully identified. Interestingly, 9 were found to have transposon insertions disrupting genes in the *cydABX* operon, which encodes cytochrome *bd*^19^,^22^ (Fig. 2A). The insertions were not only in the essential *cydA* or *cydB* gene but also in the operon promoter, implicating that the functional loss of the enzyme may dictate the suppression. As disruption of the *cydABX* operon in the screening was repeatedly identified, it is highly possible that cytochrome *bd* is associated with the growth defect of the *oxyR* mutant.

To confirm that the suppression is due to the loss of cytochrome *bd*, we deleted the entire *cyd* operon from the Δ*oxyR* strain. In *S. oneidensis*, cytochrome *bd* confers cells resistance to nitrite^22^, thus the additional mutation was validated by the finding that the Δ*oxyR*Δ*cyd* strain was sensitive to nitrite the same as the Δ*cyd* strain (Fig. 2B). As expected, the removal of cytochrome *bd* enabled the Δ*oxyR* strain to grow indistinguishably from the wild-type (Fig. 2C). In the case of the plating defect, however, the Δ*oxyR*Δ*cyd* strain was similar to the Δ*oxyR* strain (Fig. 2B). Importantly, the growth and the plating defects of the Δ*oxyR*Δ*cyd* strain were relieved, when the *cyd* operon and the *oxyR* gene were expressed *in trans*, respectively (Fig. 2B,C). These data firmly established that the loss of cytochrome *bd* suppresses the growth defect of the *oxyR* mutant.

### The *cyd* operon is up-regulated in the *oxyR* mutant

Notably, when complemented by the *cyd* operon within pHG101 the Δ*oxyR*Δ*cyd* strain grew even slower than the Δ*oxyR* strain (Fig. 2C). This is probably due to overproduction of cytochrome *bd* as the plasmid for complementation is present ~5 copies per cell^22^. Cytochrome *bd* is characterized by its high affinity for O_2_ and usually is induced under microaerobic conditions. Hence, cytochrome *bd* levels are rather low in *S. oneidensis* under normal aerobic conditions as adopted in this study^18^. Given these features, we reasoned that cellular levels of cytochrome *bd* are likely to be up-regulated in the *oxyR* mutant.

**Figure 3:**
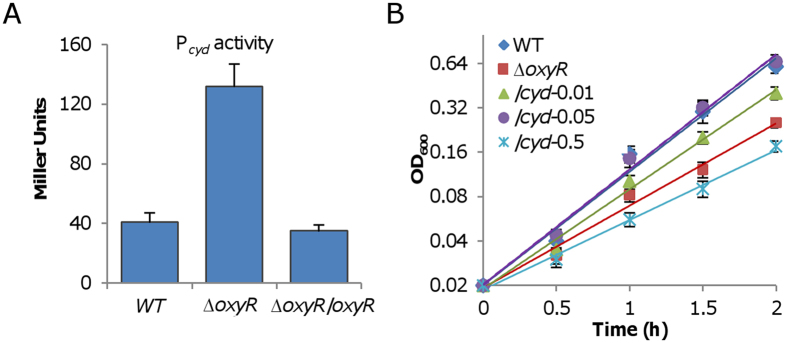
Cytochrome *bd* is in enhanced production in the *oxyR* mutant. (**A**) Promoter activity of P_*cyd*_ revealed by an integrated *lacZ* reporter in indicated strains grown in CAT-containing LB. Cells of mid-log phase cultures (~0.3 of OD_600_) were pelletted, processed, and subjected to β-galactosidase activity assay as described in Experimental procedures. (**B**) Effects of cytochrome *bd* in varying amounts on growth of ∆*oxyR*∆*cyd* in CAT-containing LB. Cytochrome *bd* was produced in cells from a copy of the *cyd* operon under control of IPTG-inducible promoter P_*tac*_. The numbers shown represent concentrations of IPTG. Experiments were performed at least three times and error bars representing standard deviation were presented.

The *cyd* promoter activity was assayed using an integrative *lacZ* reporter as before^32^. In the mid-log phase wild-type cells, the promoter activity was rather low, at levels of approximately 40 Miller units (Fig. 3A). In comparison, it increased more than three times in the *oxyR* mutant, implying that OxyR represses expression of the *cyd* operon. We then manipulated production levels of cytochrome *bd* by IPTG-controlled promoter P_*tac*_ within pHGE-Ptac to assess effect of the enzyme in varying amounts on growth of the Δ*cyd* and ∆*oxyR*Δ*cyd* strains. The expression system is highly reliable, producing protein of interest proportional to IPTG up to 1 mM^13^,^42^. Despite this, in order to precisely interpret data, levels of cytochrome *bd* proteins produced in these strains by IPTG induction were estimated by nitrite sensitivity assay. In agreement with previous calibration, the higher the IPTG level, the stronger resistance the cells to nitrite (Fig. S1).

With IPTG at 0.01 mM the ∆*oxyR*Δ*cyd* strain did not display any defect in growth (Fig. 3B). When IPTG was added to 0.05 mM, which confers the promoter ~120 Miller units according to previous calibration^13^,^42^, expression of *cyd* slowed growth of ∆*oxyR*Δ*cyd* to extent comparable to that the ∆*oxyR* strain (Fig. 3B). Growth defect became more substantial with IPTG at higher concentrations, indicating that cytochrome *bd* in excess does have a negative influence on growth.

### Loss of OxyR results in electron shortage to cytochrome *cbb*_*3*_

**Figure 4:**
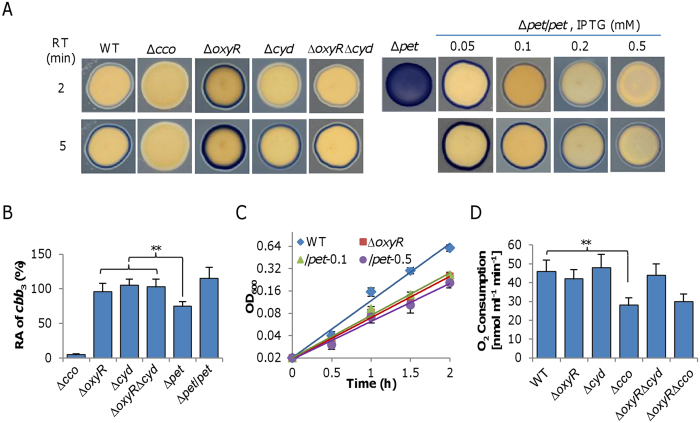
Cytochrome *cbb*_3_ suffers an electron shortage in the *oxyR* mutant. (**A**) Activities of cytochrome *cbb*_3_ in indicated strains grown on CAT-containing LB plates by the Nadi assay. The method is based on the rapid formation of indophenol blue from colorless a-naphtol catalyzed by cytochrome *c* oxidase, using *N’,N’*-dimethyl-p-phenylenediamine monohydrochloride as an exogenous electron donor. WT and previously verified ∆*cco* served as positive and negative controls. Shown on the right panel were effects of cytochrome *bc*_1_ at varying amounts on activities of cytochrome *cbb*_3_. Expression of the *pet* operon was driven by IPTG-inducible P_*tac*_ with IPTG at indicated concentrations (mM). (**B**) Activities of cytochrome *cbb*_3_ in membranes of indicated strains by the cytochrome *c* oxidase activity analysis. The *pet* mutant and its complemented strain were included for comparison. Asterisks indicate statistically significant difference (**P < 0.01). (**C**) Effects of cytochrome *bc*_1_ at varying amounts on growth of the ∆*oxyR* strain. Expression of the *pet* operon in the ∆*oxyR* strain was conducted as in (**A**) The numbers shown represent concentrations of IPTG. (**D**) Oxygen consumption rates in resting cells of the relevant strains. Oxygen consumption rates were determined as described in Methods. In (**A**) shown were representative results from multiple experiments at indicated reaction times. In others, the experiment was performed at least three times and error bars indicate standard deviation.

In *S. oneidensis*, although cytochrome *bd* is able to respire oxygen to support growth in the absence of cytochrome *cbb*_3_, its loss does not significantly affect aerobic growth^18^. Clearly, given that the *oxyR* mutation causes enhanced production of cytochrome *bd*, which promotes oxygen respiration, activity of cytochrome *bd per se* may not be a factor accountable for the growth defect caused by the OxyR loss. We therefore turned to cytochrome *cbb*_3_ because it is predominant factor for aerobic growth. We used the Nadi assay to examine activity of cytochrome *cbb*_3_ in relevant strains because it is the only enzyme reacting with Nadi reagents in *S. oneidensis*^18^. In the presence of catalase, the Δ*oxyR* strain generated a clearly visible blue ring in 2 min, stronger in intensity than that produced by the wild-type in 5 min (Fig. 4A). The negative control, a Δ*cco* strain, which lacks entire cytochrome *cbb*_3_, did not generate a blue ring after an incubation of 5 min. Thus, there appears a significant difference in cytochrome *cbb*_3_ activity between the Δo*xyR* and wild-type strains. While the loss of cytochrome *bd* (Δ*cyd*) had no impact on the activity of cytochrome *cbb*_3_ as previously reported^18^, it restored the activity of cytochrome *cbb*_3_ of Δo*xyR* to the wild-type level (Fig. 4A). It is worth noting that the additional loss of cytochrome *cbb*_3_ had an extremely weak influence on the growth defect of the *oxyR* mutant, implicating that contribution of cytochrome *cbb*_3_ to oxygen respiration in the Δ*oxyR* strain is negligible (Fig. S2).

The enhanced cytochrome *cbb*_3_ activity in the Δ*oxyR* strain detected by the Nadi reagents may be due to increased production of the enzyme. However, we found that expression levels of the *cco* operon were comparable in the wild-type, Δ*oxyR*, and Δ*oxyR*Δ*cyd* by using transcriptional fusion (Fig. S3A). The quantity of the enzyme was also assessed by measuring cytochrome *c* oxidase activity in solubilized membranes, which is independent of the cellular electron transport chain. As shown in Fig. 4B, differences in the cytochrome *cbb*_3_ activities among Δ*oxyR*, Δ*cyd*, and Δ*oxyR*Δ*cyd* strains were insignificant. Thus, we conclude that production of cytochrome *cbb*_3_ is not affected by the *oxyR* mutation.

Alternatively, it is possible that there is electron shortage to cytochrome *cbb*_3_. We have previously demonstrated that electrons from both the electron transport chain and Nadi reagents compete for cytochrome *cbb*_3_; as a result, cytochrome *cbb*_3_ is highly active with Nadi reagents when the electron transport chain is impaired^20^. The scenario can be best illustrated by a strain (∆*pet*) lacking cytochrome *bc*_1_. In *S. oneidensis*, cytochrome *bc*_1_ encoded by the *petABC* operon is responsible for transporting electrons from the quinone pool to cytochrome *cbb*_3_, and its loss results in a growth defect comparable to that of the Δ*cco*^32^. Consistent with our previous data^20^, the loss of cytochrome *bc*_1_ drastically increased the activity of cytochrome *cbb*_3_ based on the Nadi assay, with the whole colony turning blue in 2 min (Fig. 4A). However, this was not observed in disrupted cells, in which the *cbb*_3_ activity of the *pet* mutant was actually lower than that of the wild-type (Fig. 4B). This is not surprisingly because the loss of cytochrome *bc*_1_ down-regulates expression of the *cco* genes^18^.

One possibility for reduced electron transfer to cytochrome *cbb*_3_ is that cytochrome *bc*_1_ is produced less in the Δ*oxyR* strain. But the promoter activity of the *pet* operon did not significantly change in Δo*xyR* comparing to that of the wild-type (Fig. S3A). We then made attempts to manipulate production of cytochrome *bc*_1_ such that the electron flow to cytochrome *cbb*_3_ can be altered. The *pet* operon was placed under the control of the IPTG– induced P_*tac*_ promoter within pHGE-P*tac* and the resulting vector was introduced into the *pet* mutant. As expected, the cytochrome *cbb*_3_ activity revealed by the Nadi assay decreased with IPTG levels inversely (Fig. 4A), implicating that electrons delivered to cytochrome *cbb*_3_ increase in quantity when cytochrome *bc*_1_ is overproduced, albeit not necessarily in a proportion manner. Despite this, overproduction of cytochrome *bc*_1_ with IPTG up to 0.5 mM had no effect on correcting the growth defect of the Δ*oxyR* strain (Fig. 4C).

All of these observations suggest that in the Δ*oxyR* strain the majority of electrons are transferred to cytochrome *bd* whose production is enhanced, resulting in low efficacy of oxygen respiration. If so, we would expect that oxygen consumption in the *oxyR* mutant may not be significantly altered. Indeed, the *oxyR* mutant had an oxygen consumption rate comparable to the wild-type, Δ*cyd*, and Δ*oxyR*Δ*cyd* which was about 90% higher than the strain lacking cytochrome *cbb*_3_ (Fig. 4D). The additional removal of OxyR in the *cco* mutant did not improve the oxygen consumption rate. This is conceivable given that cytochrome *bd* is overproduced in the absence of cytochrome *cbb*_3_^18^. Hence, the suppression of the Δ*oxyR* growth defect by the loss of cytochrome *bd* is likely due to that more electrons from the quinone pool are delivered to cytochrome *cbb*_3_ as a result of the shutdown of the alternative route.

### Lack of CymA partly rescues the growth defect of the *oxyR* mutant

Given that the *bc*_1_ complex in overabundance did not influence the electron flow toward cytochrome *cbb*_3_, we proposed the quinone pool as the next candidate responsible for the growth defect of Δo*xyR*. If so, it is possible that other quinone oxidases, at least the major one, in their absence may also suppress the growth defect resulting from the *oxyR* mutation. In *S. oneidensis*, the quinol pool passes electrons to many quinone oxidases, including CymA, TorC, SirCD, PsrBC, cytochrome *bc*_1_, as well as cytochrome *bd*^30^,^43^,^44^,^45^. While CymA is linked to respiration of a variety of non-oxygen EAs, TorC, SirCD, and PsrBC are specific for TMAO, sulfite, and thiosulfate respiration, respectively. Moreover, CymA but not TorC, SirCD, or PsrBC is produced considerably in the presence of oxygen^30^,^46^,^47^, implying that CymA may be capable of consuming electrons from the quinone pool under aerobic conditions.

**Figure 5:**
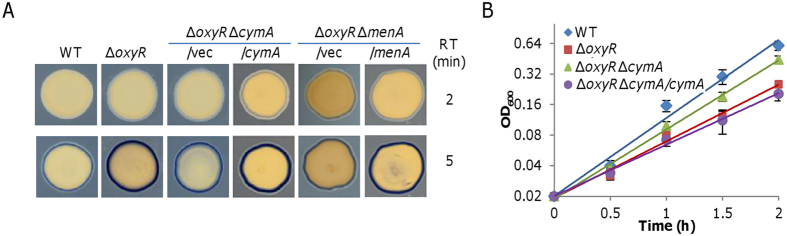
CymA is associated with the growth defect of the *oxyR* mutant. (**A**) Activities of cytochrome *cbb*_3_ in indicated strains grown on CAT-containing LB plates by the Nadi assay. Shown were representative results from multiple experiments at indicated reaction times. (**B**) Growth of indicated strains in CAT-containing LB. Experiments were performed at least three times with error bars representing standard deviation.

To test this, we deleted *cymA* from the Δo*xyR* strain. The Nadi assay revealed that Δo*xyR*Δ*cymA* generated a blue ring by a significantly slower rate than the Δo*xyR* strain, but still faster than the wild-type did (Fig. 5A), supporting an alleviation of the electron shortage for the cytochrome *cbb*_3_. Consistently, the growth defect of Δo*xyR* was partially corrected by the additional removal of *cymA* (Fig. 5B). These data suggest that CymA and cytochrome *bd* likely function in a similar manner in regard of the growth defect of Δo*xyR*, and strongly support that electron deficiency for cytochrome *cbb*_3_ is the main reason for the growth defect of Δo*xyR*. Although EA-specific quinone oxidases (TorC, SirCD, and PsrBC) are barely produced under test conditions, we removed each from the Δo*xyR* strain and assessed impacts of the additional removal on the growth defect. Expectedly, all resulting double mutants were indistinguishable from Δo*xyR* with respect to growth (data not shown), ruling out the possibility that these proteins play an indispensable role in consuming electrons from the quinone pool under test conditions. Importantly, production of CymA in the Δo*xyR* strain was similar to that in the wild-type (Fig. S3B), indicating that the electron shortage to cytochrome *cbb*_3_ was not due to excessive CymA. All together, these results indicate that quinone oxidase CymA has ability to draw electrons from the quinone pool under test conditions, contributing to an electron shortage for cytochrome *cbb*_3_.

### The ratio of MKs to UQs increases in the *oxyR* mutant

**Figure 6:**
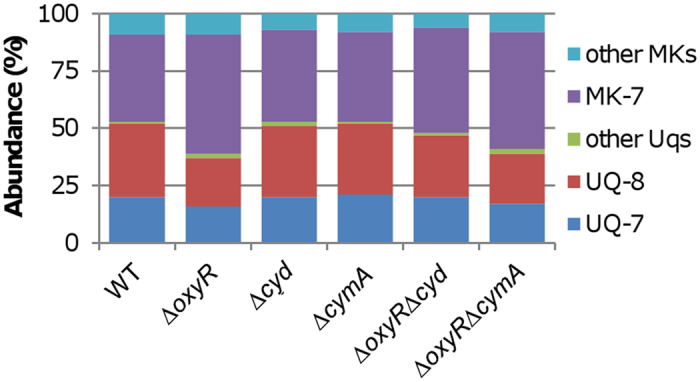
UQs/MKs ratio is reduced in the *oxyR* mutant. UQ/MK profiles in indicated strains were compared. Cells of mid-log phase cultures (~0.3 of OD_600_) grown in CAT-containing LB were pelletted, processed, and subjected to Quinone extraction as described in Experimental procedures. Quinone identification and quantification by LC-MS were described in the Experimental procedure and text. Experiments were performed at least three times and standard variations were within 5% range.

Results presented thus far suggest that respiratory quinones are likely a key factor for the growth defect resulting from the *oxyR* mutation. Given that loss of either cytochrome *bd* or CymA improves Δo*xyR* growth, it is possible that electron deficiency for cytochrome *cbb*_3_ in Δo*xyR* is due to the changes in the composition of quinones. To test this possibility, we determined levels of quinones from the wild-type, Δ*oxyR*, Δ*cyd*, Δ*cymA*, Δ*oxyR*Δ*cyd*, and Δ*oxyR*Δ*cymA* strains grown to the mid-log phase under aerobic conditions with high-performance liquid chromatography (HPLC) and mass spectrometer (MS) (Fig. 6). The ratio of UQs to MKs in the wild-type was approximately 52:48 under test conditions, which is in excellent agreement with previous data^27^,^32^. Similar results were obtained from Δ*cyd* and Δ*cymA*, indicating that the loss of either gene alone would not significantly affect production of UQs and MKs. In contrast, the ratio was about 43:57 in the Δ*oxyR* strain, suggesting that the *oxyR* mutation results in substantially changes in production of these quinones. Strikingly, the additional removal of cytochrome *bd* in the *oxyR* mutant had a significant influence (*p* < 0.05) on the ratio, restoring it (48:52) to levels close to that of the wild-type. In contrast, the depletion of CymA had no such effect. These data suggest that cytochrome *bd* functions more than a competing quinone oxidase for electrons against cytochrome *bc*_1_ (for cytochrome *cbb*_3_).

### Disruption of MK biosynthesis partially rescues the growth defect of the *oxyR* mutant

**Figure 7:**
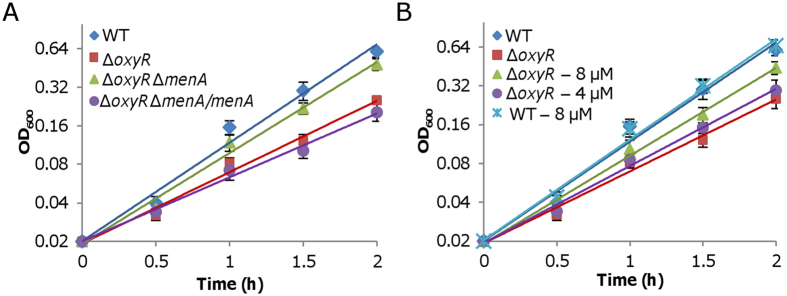
Suppression of ∆*oxyR* growth defect by altered levels of UQs and MKs. (**A**) Growth of indicated strains in CAT-containing LB. (**B**) Growth of indicated strains in CAT-containing LB with UQ-10 addition. UQ-10 concentrations used in the experiment were shown. Experiments were performed at least three times with error bars representing standard deviation.

To confirm that the ratio of UQs to MKs is linked to the *oxyR* mutant growth phenotype, we assessed effects of the *menA* mutation on the growth defect of the *oxyR* mutant^32^. The *menA* gene encodes 1,4-dihydroxynahthoate (DHNA) octaprenyltransferase, an essential component of MK-7 biosynthesis pathway that converts DHNA to dimethylmenaquinone^48^. The Δ*oxyR*Δ*menA* strain grew apparently faster than Δ*oxyR* did, at a rate similar to that of Δ*oxyR*Δ*cymA* (Fig. 7A). Consistently, cytochrome *cbb*_3_ activities based on the Nadi reaction in Δ*oxyR*Δ*menA* and Δ*oxyR*Δ*cymA* were also comparable (Fig. 5A). The same analysis could not be applied to UQ biosynthesis, because we failed to knock out any gene essential for UQ biosynthesis after many tries^32^. Instead, we performed chemical complementation. In the presence of UQ (Q10) up to 8 μM, growth of the wild-type was not affected (Fig. 7B). In contrast, an improvement in growth of Δ*oxyR* was observed with UQ at 4 and 8 μM, suggesting that exogenous UQ can partially correct the growth defect resulting from the *oxyR* mutation.

A remaining possibility that could address the growth defect of the *oxyR* mutant is that electrons entering the quinone pool are reduced in quantity. To test this, we monitored the NAD^+^/NADH ratio as these redox chemicals are predominant electron carriers, especially for aerobic respiration. However, the difference between the wild-type and Δ*oxyR* strains was insignificant (Fig. S4A). In addition, we compared in these two strains the promoter activities of the *nuo* operon, which encodes NADH dehydrogenase that couples the electron transfer from NADH to quinones with a proton translocation^49^. Similarly, the results were comparable (Fig. S4B). By ruling out the difference in electrons entering the quinone pool, the data conclude that the ubiquinone/menaquinone levels, which are altered in the *oxyR* mutant, are associated with the growth defect.

### OxyR represses cytochrome *bd* and MK production in an indirect manner

**Figure 8:**
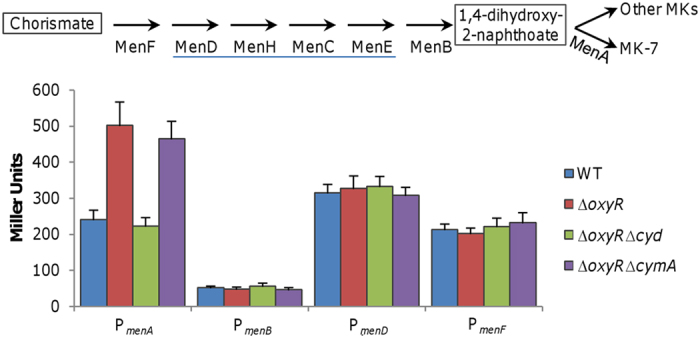
Expression of *menA* is enhanced in the *oxyR* mutant. Activity of promoters for MK-7 biosynthesis operons were assayed by an integrated *lacZ* reporter in indicated strains grown in CAT-containing LB. The MK-7 biosynthesis pathway was outlined. MenDHCE, underlined, are predicted to be co-transcribed (www.biocyc.org). Cells of mid-log phase cultures (~0.3 of OD_600_) were prepared the same as in Fig. 3A. Experiments were performed at least three times with error bars representing standard deviation.

To explain why the ratio of UQs to MKs decreases in the *oxyR* mutant, we analyzed the activity of promoters for some *ubi* (UQ biosynthesis) and *men* (MK biosynthesis) genes. In line with the unchanged levels of UQs in the wild-type and Δ*oxyR* strains, activities of promoters for all *ubi* operons were not significantly affected by the *oxyR* mutation (data not shown). The MK biosynthetic pathway entails 7 reactions that convert chorismate to MK-7 by 7 genes in 4 predicted operons, MenF, MenDHCE, MenB, and MenA (Fig. 8). Although most *men* promoters were unresponsive to the *oxyR* mutation the *menA* promoter displayed enhanced activity, approximately 2-fold relative to that in the wild-type (Fig. 8). Because MenA is specific for MK-7 synthesis while all other Men proteins are responsible for production of 1,4-dihydroxy-2-naphthoate, an intermediate for all MKs, the result supports that MK-7 is probably only MK species in increased production in the *oxyR* mutant as revealed above (Fig. 6).

OxyR is regarded as a global regulator but its regulon remains poorly defined in *S. oneidensis*^12^. To determine whether OxyR regulates *cyd* and *menA* transcription in a direct manner, we employed bacterial one-hybrid (B1H) to assess the interaction between OxyR and the *menA* promoter sequence. The B1H system used here is particularly effective and efficient as it is derived from the BacterioMatch II two-hybrid system^41^. Vectors containing ‘bait’ (DNA) and ‘target’ (OxyR) were co-transformed into the reporter strain, of which those having positive DNA-protein interactions are able to grow on selective plates. The reporter strains carrying positive control pair P_*katB*_ and OxyR (OxyR in reduced form repressing the *katB* gene) formed a large number of colonies on selective plates (Table 2). A similar result was obtained with the reporter strains carrying another positive control pair P_*ahpC*_ and OxyR^L197P^, an OxyR mutant that is locked in the activated state for transcription of the *ahpC* gene^50^. In contrast, the reporter strain having negative control pair (P_16s-rRNA_/OxyR and P_16s-rRNA_/OxyR^L197P^) developed a few colonies, which, importantly, were unable to grow on confirmation plates. When P_*cyd*_/OxyR, P_*cyd*_/OxyR^L197P^, P_*menA*_/OxyR, and P_*menA*_/OxyR^L197P^, were under examination, the reporter strains failed to form any colonies that can pass through confirmation. These data suggest that the regulatory region of the *menA* gene unlikely interacts with OxyR in *S. oneidensis*.

## Discussion

In many bacteria, OxyR is now regarded as a pleiotropic regulator and its loss causes multiple defects in various biological processes^1^,^51^. While some are species-dependent, two are profound and common: viability deficiency and growth defect. This is not surprising as most OxyR proteins studied to date function as an activator, at least for H_2_O_2_–scavenging genes. Because it seems logic to attribute these two defects of *oxyR* mutants to reduced ability to scavenge endogenous H_2_O_2_, direct experimental validation lags behind, if not totally overlooked. The scenario is different in *S. oneidensis*, whose OxyR acts as a repressor for major catalase gene *katB* such that KatB levels in the *oxyR* mutant are high, leading to rapid H_2_O_2_ degradation^12^. Thus, we were surprised when we found that the *S. oneidensis oxyR* mutant also carries these defects. After illustrating the mechanism accountable for viability deficiency^13^, we made efforts to identify factors underlying the growth defect of the *oxyR* mutant.

**Figure 9:**
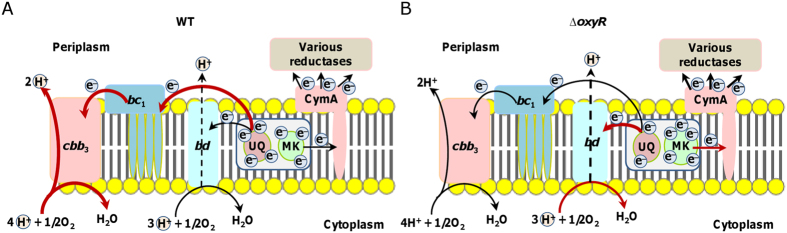
Model illustrating the mechanism underlying the growth defect of the *oxyR* mutant. The electron transport chain in the cytoplasmic membrane links the quinone pool with cytochrome *cbb*_3_, cytochrome *bd*, and various terminal reductases. All relevant components were included. The quinone pool is composed of UQs and MKs, which act as electron donors specifically for terminal oxidases (cytochrome *cbb*_3_ and *bd*) and CymA, respectively. (**A**) In the wild-type, UQs are slightly more abundant than MKs. The majority of electrons (by thick red lines) in the quinone pool are delivered to energy-efficient cytochrome *cbb*_3_ for oxygen respiration. (**B**) In the *oxyR* mutant, cytochrome *bd* is overproduced, impairing energy conservation (proton gradient) because of its low efficiency (dash line represents non-proton-pump mechanism for proton translocation) and low activity of cytochrome *cbb*_3_ due to an electron shortage. In addition, the UQs/MKs ratio is reduced, leading to less electrons available for oxygen respiration. The terminal phenotype for the *oxyR* mutant is reduced growth rate.

Viability deficiency is largely due to cellular damages caused by ambient H_2_O_2_, which is spontaneously generated in rich media^13^. As exogenous catalase is able to fully suppress this defect but fails in correcting the growth defect, it is apparent that different factors are responsible for the latter. Upon revelation of reduced electron supply from the quinone pool to cytochrome *cbb*_3_ in the *oxyR* mutant as a main cause to the growth defect, we suggest a model to explain what we have observed in this study (Fig. 9). In *S. oneidensis*, cytochrome *cbb*_3_, which obtains electrons from ubiquinones via cytochrome *bc*_1_, is the primary system for oxygen respiration, and thus dictates aerobic growth^18^. Although abundance of both cytochrome *cbb*_3_ and cytochrome *bc*_1_ is not affected by the *oxyR* mutation, fewer electrons are passed to these protein complexes, resulting in growth defect of the *oxyR* mutant. Quantities of electrons in the quinone pool may not be significantly altered, based on the observation that the mutation has a negligible impact on the NAD^+^/NADH ratio. Instead, substantial increase in production of cytochrome *bd* and MK-7, especially the former, leads to drastic reduction in the number of electrons that are passed to cytochrome *cbb*_3_. By removing other quinone oxidases, cytochrome *bd* and CymA, electron flow to cytochrome *bc*_1_ augments, leading to suppression of the growth defect.

In the electron transport chain for respiration, the quinone pool plays a central role as a recyclable electron reservoir which accepts electrons from donors such as NADH and passes them to quinone oxidases, and eventually to electron accepting molecules^25^. This is particularly important for *S. oneidensis*, in which respiration is the only means of energy generation to support growth^52^. Given that *Shewanella* thrive in redox-stratified environments, evolution has arranged to produce UQs and MKs in considerable quantities under any circumstance and at least three quinone oxidases, cytochrome *bd*, cytochrome *bc*_1_, and CymA during aerobiosis, such that simultaneous respiration of multiple EAs could proceed. For example, nitrate is reducible by cells at any growth phase under aerobic conditions and many EAs can be respired after aerobic-growing cells enter the stationary phase^53^,^54^. In the *oxyR* mutant, the composition of UQs and MKs is altered significantly, largely due to increased levels of MKs. Given that UQs are preferable, if not exclusive, electron donors for oxygen respiration, reduction in UQ contents, or at least in proportion, diminishes electron flow to cytochrome *bc*_1_ and then to cytochrome *cbb*_3_, resulting in the growth defect. In line with this, the defect is relieved by either depleting MKs or adding exogenous UQ.

Under aerobic conditions, oxidative phosphorylation, the predominant way for the conservation of energy, is linked to an energized state of the membrane, which is established by electron transport reactions between electron carriers^25^. The process is initiated with NADH, a molecule generated mainly through the tricarboxylic acid (TCA) cycle. NADH dehydrogenase transfer electrons from NADH (oxidation) to quinones such as UQs or MKs. This electron input route of *S. oneidensis* into the quinone pool largely resembles that established in the model organisms. More importantly, the electron input into the quinone pool is hardly affected by the *oxyR* mutation based on the finding that the intracellular ratio of NAD^+^ to NADH is unchanged in the *oxyR* mutant. In contrast, the electron output from the quinone pool is carried out by remarkably diverse electron transport pathways^20^,^30^,^32^. As a consequence, a competition exists among quinone oxidases that couple the quinone pool to different electron accepting molecules. Among three quinone oxidases produced during aerobiosis, CymA not only requires MK-7 as a co-factor but also has a clear catalytic bias towards the reduction of MK-7^28^,^30^. Moreover, given the reduction potentials of CymA and UQs, oxidation of UQs by CymA is thermodynamically unfeasible^30^. Hence, it is not surprising that the impact of the CymA loss on the growth defect of the *oxyR* mutant is rather limited. In contrast, there is direction competition between cytochromes *bd* and *bc*_1_ for electrons carried by UQs. When the ratio of cytochromes *bd* to UQs increases, less electrons are transferred to cytochrome *bc*_1_. Our data also suggest that cytochrome *bd* is advantageous of cytochrome *bc*_1_ in reducing UQs. Overproduction of cytochrome *bd* worsens its inhibitory effect on the growth of the *oxyR* mutant, but cytochrome *bc*_1_ in excess could not alleviate the defect. This observation is in line with the differences in oxygen affinity between cytochrome *bd* and *cbb*_3_; cytochrome *bd* has a higher affinity to oxygen than cytochrome *cbb*_3_. As such, when cytochrome *bd* is produced, it consumes electrons in the quinone pool to respire oxygen over cytochrome *cbb*_3_. To compete under aerobic conditions, *S. oneidensis* tends to repress production of cytochrome *bd* in the presence of cytochrome *cbb*_3_^18^. Derepression can be achieved when cytochrome *cbb*_3_ is depleted or cells grow in certain conditions, such as in the presence of nitrite or cAMP at elevated levels^22^,^23^.

UQs appear to be essential to aerobic respiration in *S. oneidensis*. This is not surprising because the bacterium that is unable to ferment^52^. Although levels of UQs in the *oxyR* mutant do not change significantly, the ratio of UQs to MKs is substantially reduced due to enhanced production of the latter. Commonly, only a few steps, especially those rate-limiting ones, in a biosynthesis pathway are conditionally inducible, such as *hemA, hemF*, and *hemH* in heme synthesis^23^,^55^. In line with this, the presented data demonstrate that *menA*, out of 7 *men* genes for the MK biosynthetic pathway^56^, is only one subjected to repression by OxyR. Coincidently, the *cyd* operon is also under repression of OxyR. The repression, however, appears indirect because OxyR fails to interact with the *menA* promoter region. Like the *cyd* operon^18^,^22^, the *menA* gene is probably to be directly regulated by cAMP-Crp as a conserved Crp-binding motif (5’-ATCTGTGCGCCAATTCAAACTC) resides in the upstream region^57^. In *E. coli*, a key physiological role of the cAMP-Crp complex is to ensure resources to be spent on distinct metabolic sectors as needed in different nutrient environments^58^. But this may not be the case in *S. oneidensis* given that the cAMP-Crp complex critically deviates from the *E. coli* paradigm with respect to regulons^57^. Nevertheless, given the consistent responses of the *menA* gene and the *cyd* operon to the *oxyR* mutation, it is likely that OxyR plays a role in mediating respiration. Efforts to test this notion are underway.

Why does the *oxyR* mutation up-regulate production of cytochrome *bd* and MKs ? Given that the ultimate consequence is the reduced growth rate, we propose that the *oxyR* mutant probably exploits both to diminish production of endogenous ROS. There is a large body of evidence to show that cytochrome *bd* enhances bacterial resistance to oxidative stress^24^. Although not completely understood, two mechanisms underpinning it are proposed: cytochrome *bd* may act as an O_2_-scavenger and electron sink as well as ROS metabolizer^59^,^60^,^61^. Due to its high affinity for O_2_ and low energetic efficiency, cytochrome *bd* is able to reduce the intracellular O_2_ levels without generating sufficient energy to support fast growth, and thus repress ROS formation, H_2_O_2_ in particular. Moreover, ROS formation is also repressed by lowered electron availability for ROS-generation enzymes because electrons in the quinone pool are simultaneously dissipated. As a result, although the *oxyR* mutant grows at a reduced rate, its ability to consume oxygen is not significantly compromised. On the other hand, cytochrome *bd* can directly remove H_2_O_2_ and other ROS^62^. But this may not be critical in *S. oneidensis* because the *oxyR* mutant has elevated ability to remove H_2_O_2_, which overshadows the ROS-scavenger activity of cytochrome *bd*^12^,^13^. In comparison with cytochrome *bd*, which is no doubts the major system accountable for the growth defect of the *oxyR* mutant, MKs seem auxiliary. The Increased levels of MKs resulting from the *oxyR* mutation may prompt dissipation of electrons onto non-oxygen EAs, leaving less to react with oxygen, thus limiting growth rate and ROS formation.

## Additional Information

**How to cite this article:** Wan, F. *et al*. Loss of OxyR reduces efficacy of oxygen respiration in *Shewanella oneidensis. Sci. Rep.* **7**, 42609; doi: 10.1038/srep42609 (2017).

**Publisher's note:** Springer Nature remains neutral with regard to jurisdictional claims in published maps and institutional affiliations.

## Supplementary Material

Supplementary Information

## Figures and Tables

**Table 1:** Strains and plasmids used in this study.

Strain or plasmid	Description	Reference or source
*E. coli* strains		
DH5α	Host for cloning	Lab stock
WM3064	Δ*dapA*, donor strain for conjugation	W. Metcalf, UIUC
*S. oneidensis* strains		
MR-1	Wild type	Lab stock
HG0483	∆*sirC* derived from MR-1	32
HG0608-10	∆*pet* (∆*petABC*) derived from MR-1	32
HG1233	∆*torC* derived from MR-1	33
HG1910	∆*menA* derived from MR-1	32
HG1328	∆*oxyR* derived from MR-1	12
HG1328-CYD	∆*oxyR*∆*cyd* derived from HG3286-4	This study
HG1328-CCO	∆*oxyR*∆*cco* derived from HG2364-1	This study
HG1328-CYM	∆*oxyR*∆*cymA* derived from HG4591	This study
HG1328-MENA	∆*oxyR*∆*menA* derived from HG1910	This study
HG1328-SIRC	∆*oxyR*∆*sirC* derived from HG0483	This study
HG1328-TORC	∆*oxyR*∆*torC* derived from HG1233	This study
HG1328-PSRC	∆*oxyR*∆*psrC* derived from HG4060	This study
HG2364-1	∆*cco* (∆*ccoNOQP*) derived from MR-1	20
HG3286-4	∆*cyd* (∆*cydABX*) derived from MR-1	20
HG4060	∆*psrC* derived from MR-1	47
HG4591	∆*cymA* derived from MR-1	47
HG1328S	Suppressor strains derived from HG1328	This study
Plasmid		
pHGM01	Ap^r^, Gm^r^, Cm^r^, *att*-based suicide vector	33
pHG101	Km^r^, promoterless broad-host vector	34
pHGE-Ptac	Km^r^, IPTG-inducible expression vector	35
pHGEI01	Km^r^, integrative *lacZ* reporter vector	32
pBBR-Cre	Sp^r^, helper plasmid for antibiotic cassette removal	22
pHGT01	Gm^r^, Promoter-embedded transposon vector	20
pBXcmT	B1H bait vector	41
pTRG	B1H target vector	Strategene
pHGEI01-P*pet*	P*pet*-*lacZ* fusion within pHGEI01	32
pHGEI01-P*cyd*	P*cyd*-*lacZ* fusion within pHGEI01	63
pHGEI01-P*cco*	P*cco*-*lacZ* fusion within pHGEI01	63
pHGEI01-P*cymA*	P*cymA*-*lacZ* fusion within pHGEI01	This study
pHGEI01-P*nuo*	P*nuo*-*lacZ* fusion within pHGEI01	This study
pHGEI01-P*menA*	P*menA*-*lacZ* fusion within pHGEI01	This study
pHGEI01-P*menB*	P*menB*-*lacZ* fusion within pHGEI01	This study
pHGEI01-P*menD*	P*menD*-*lacZ* fusion within pHGEI01	This study
pHGEI01-P*menF*	P*menF*-*lacZ* fusion within pHGEI01	This study
pHGE-Ptac-*cyd*	Vector for inducible expression of *cyd*	This study
pHGE-Ptac-*pet*	Vector for inducible expression of *pet*	This study
pBXcmT-16s-rRNA	B1H bait vector carrying promoter for 16s-rRNA gene	12
pBXcmT-*katB*	B1H bait vector carrying *katB* promoter	12
pBXcmT-*ahpC*	B1H bait vector carrying *ahpC* promoter	12
pBXcmT-*cyd*	B1H bait vector carrying *cyd* promoter	12
pBXcmT-*menA*	B1H bait vector carrying *menA* promoter	This study
pTGR-*crp*	B1H target vector producing Crp	12
pTRG-*oxyR*	B1H target vector producing OxyR	12
pTRG-*oxyR*^*T613C*^	B1H target vector producing OxyR^L197P^	This study

**Table 2:** Bacterial one-hybrid (B1H) assay of OxyR with the *menA* promoter.

Bait Vector pBXcmT	Target Vector pTRG	No. of colonies on nonselective plates^a^	No. of colonies on selective plates^b^	Confirmation^c^
/—	/—	>500	0	—
/P_*katB*_	/OxyR	>500	187	184
/P_16S-rRNA_	/OxyR	>500	2	0
/P_*ahpC*_	/OxyR^L197P^	>500	165	158
/P_16S-rRNA_	/OxyR^L197P^	>500	3	0
/P_*menA*_	/OxyR	>500	1	0
/P_*menA*_	/OxyR^L197P^	>500	1	—

^a^M9 agar +25 μg/ml chloramphenicol +12.5 μg/ml tetracycline. ^b^a + 5 mM 3-AT. ^c^b + 12.5 μg/ml streptomycin.
