# Supplementary material for: Loss of OxyR reduces efficacy of oxygen respiration in Shewanella oneidensis
Source: Sci Rep. 2017 Feb 14;7:42609. doi: 10.1038/srep42609 (PMC5307378; doi:10.1038/srep42609)
Supplement: Supplementary Information [file srep42609-s1.pdf]

Supplemental materials of

Loss of OxyR reduces efficacy of oxygen respiration in *Shewanella oneidensis*

Fen Wan, Miaomiao Shi, and Haichun Gao*

Institute of Microbiology and College of Life Sciences, Zhejiang University, Hangzhou, Zhejiang, 310058, China

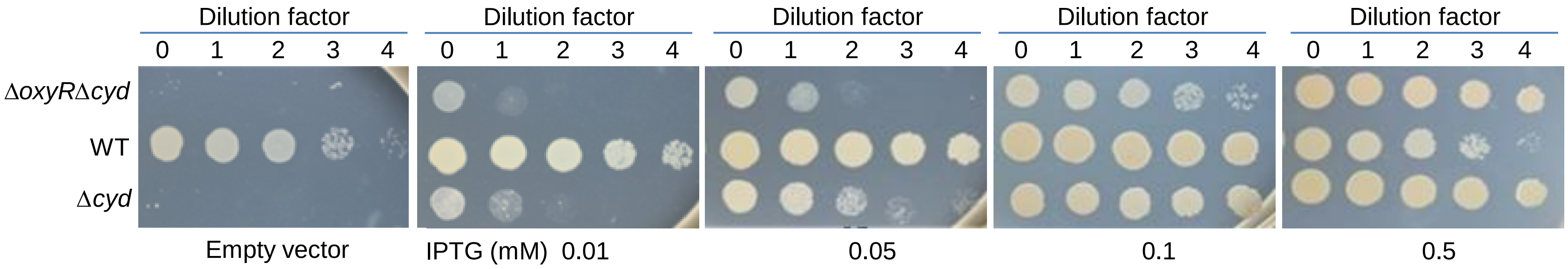

Fig. S1. Calibrating expression levels of *cyd* in *S. oneidensis*. The *cyd* operon was placed under the control of P_{tac} within pHGE-Ptac and the resulting vector was introduced into strains indicated. Production levels of cytochrome *bd* were estimated by resistance to nitrite. Strains carrying empty vector were used as the control. The assay was performed on LB plates containing 4 mM nitrite with IPTG at indicated concentrations.

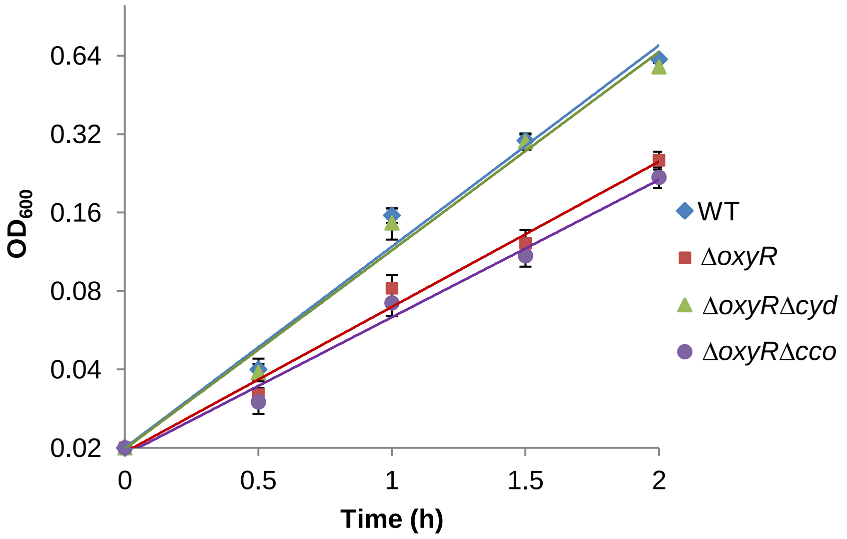

Fig. S2. Effects of the cytochrome *cbb*$_3$ loss on growth of Δ*oxyR* in CAT-containing LB. Experiments were performed at least three times and error bars representing standard deviation.

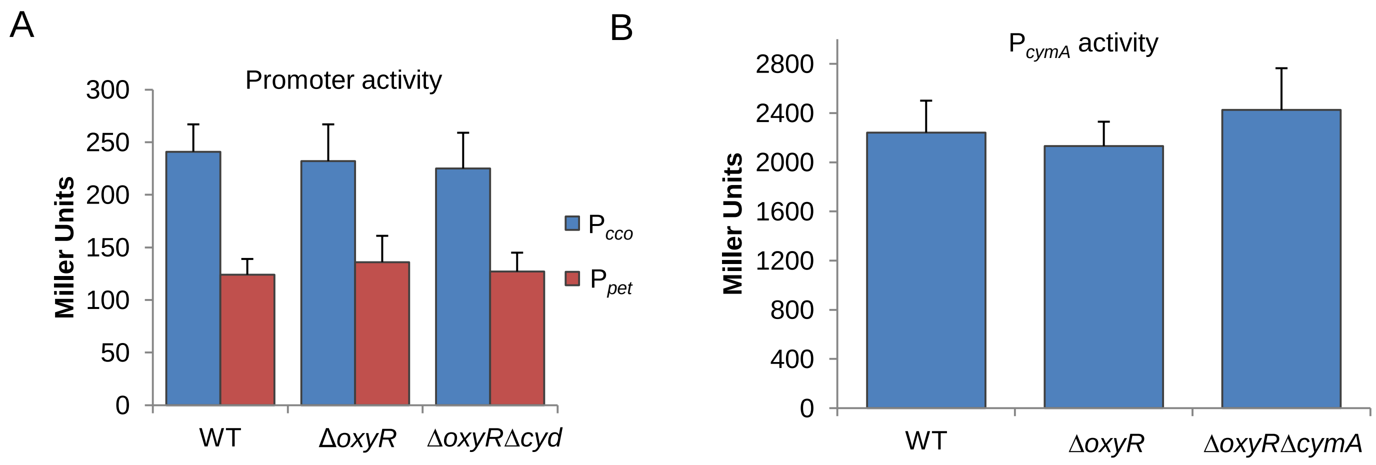

Fig. S3. Promoter activities in the *oxyR* mutant. A. Promoter activity of indicated promoters revealed by an integrated *lacZ* reporter in indicated strains grown in CAT-containing LB. **B.** Promoter activity of indicated promoters revealed by an integrated *lacZ* reporter in indicated strains grown in CAT-containing LB. Cells of mid-log phase cultures (~0.3 of OD_{600}) were pelletted, processed, and subjected to β-galactosidase activity assay as described in Experimental procedures. Experiments were performed at least three times and the average ± error bars representing standard deviation were presented.

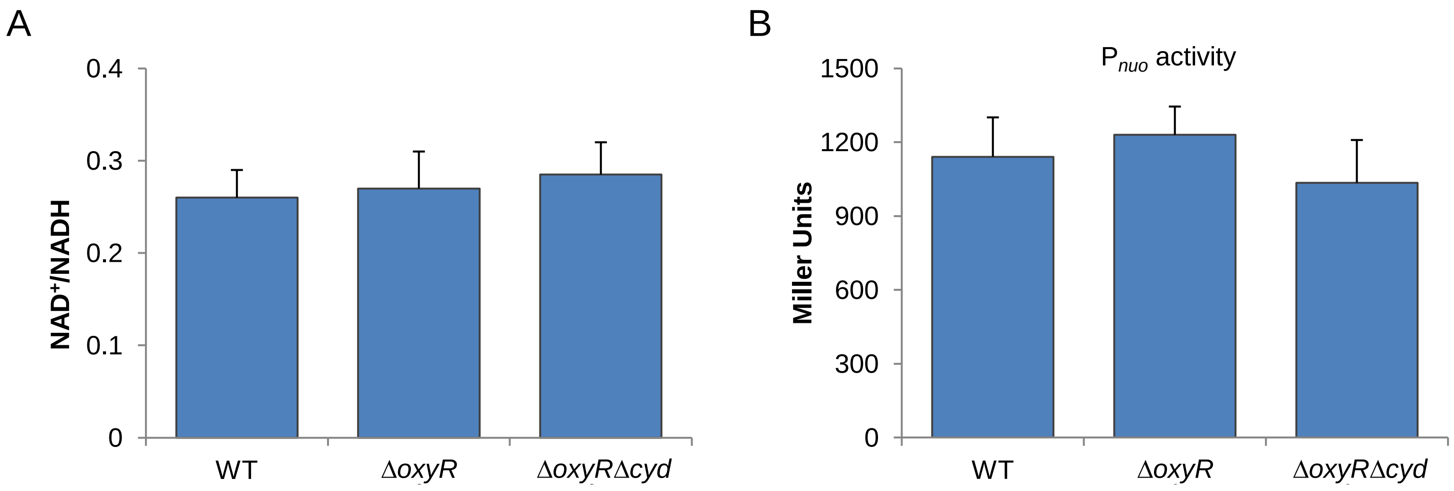

Fig. S4. Biology regarding electron input into the quinone pool in *S. oneidensis* strains. A. The ratio of NAD^+ to NADH in indicated strains grown to the mid-log phase in CAT-containing LB. **B.** Promoter activity of indicated promoters revealed by an integrated *lacZ* reporter in indicated strains grown in CAT-containing LB. Cells of mid-log phase cultures (~0.3 of OD_{600}) were pelletted, processed, and subjected to β-galactosidase activity assay as described in Experimental procedures. Experiments were performed at least three times and the average ± error bars representing standard deviation were presented.
